# Disordered Eating and Eating Disorders in Adolescent Athletes

**DOI:** 10.51894/001c.11595

**Published:** 2020-01-30

**Authors:** Ryley Mancine, Samantha Kennedy, Peter Stephan, Alyse Ley

**Affiliations:** 1 Michigan State University College of Osteopathic Medicine; 2 Department of Psychiatry Michigan State University College of Osteopathic Medicine

**Keywords:** disorderd eating, female athlete triad, adolescent, athlete

## Abstract

**CONTEXT:**

To summarize available literature to date and discuss the importance of Disordered Eating (DE) in adolescent athletes, with special attention to the female athlete triad. In this paper, the authors will review the literature regarding adolescent athletes who intentionally engage in abnormal eating behaviors and focus on adolescent athletes of all training levels who may be affected by both DE and eating disorders (ED).

**METHODS:**

In 2019, the authors completed a systematic literature search on PubMed using the search term variations of “Feeding and Eating Disorders” and “athletes” with “high school.”

**RESULTS:**

A total of 20 pertinent articles were identified concerning DE in adolescent athletes. ED have been shown to impose higher rates of comorbidity than other psychological disorders and only a small number of individuals with ED seek treatment. ED tend to be more prevalent in adolescent elite athletes than non-athletes of both genders in all sports and levels of competition.

**CONCLUSIONS:**

More rigorous tools for family practice physicians, nurses, and coaches to use when working with at-risk adolescent athletes are needed to identify DE behaviors. Healthcare and school professionals need to be educated and trained to detect DE and the components of the female athlete triad. Additional research with adolescent males or those associating with alternative gender roles is also required to help them prevent physical and mental health consequences associated with DE.

## INTRODUCTION

There are currently over 4.5 million male and 3.4 million female high school athletes in the United States.^1^ For many students, participation in a competitive high school sport has a positive impact, including higher grades and more positive attitudes toward schooling.^2^ In fact, participation in high school sports is the most significant predictor of physical activity in later life for many males.^3^ Unfortunately, some student athletes’ participation in high school sports can lead to unintended health consequences. Potential issues include increased risk of stress fracture as well as emotional and mood instability.^4^

The female athlete triad, which has been defined by the Female Athlete Triad Coalition as a combination of disordered eating (DE), amenorrhea (i.e., lack of menstruation) , and osteoporosis, can affect female athletes at all levels of competition and all sports and may have severe health consequences.^5,6^ DE is defined as potentially harmful or disruptive eating behaviors and is a term most frequently seen in research, while Eating Disorders (ED) are true psychiatric diagnoses made using the Diagnostic and Statistical Manual of Mental Disorders, Fifth Edition (DSM-5).^7,8^ DE encompasses a subclinical spectrum of pathological eating behaviors and may lead to ED.^8^ It has been recommended that individuals experiencing the female athlete triad should undergo a three-step decision-based model before returning to competition. Male athletes can also develop DE habits.^9–11^

Additionally, insufficient caloric intake in young athletes can unintentionally occur in higher-level adolescent athletes.^5^ When adolescent athletes transition to higher levels of competition, the number of weekly training hours typically increases. This increase in training volume coupled with inadequate knowledge of proper nutrition can result in an energy imbalance.^5^ Although the negative thoughts of DE and ED may not be present, there can still be negative health impacts. Those caring for athletes’ health need to monitor their caloric intake and provide appropriate nutritional counseling.^4^

Although the research focusing on adolescent male athletes has been relatively sparse, studies including adolescent males has demonstrated that they are also at risk for DE behaviors.^12^ Whereas female DE behaviors have been shown to primarily focus on weight loss, male DE behaviors may tend to focus on muscle gain, with an increased risk for steroid use.^12^ DE behaviors including restrictive eating, meal skipping, fasting, usage of diet pills, and other behaviors can be used by both males and females to alter body weight or shape.^13,14^

Research has demonstrated an increased incidence of disordered eating (DE) in male athletes compared to non-athletic counterparts, though many studies with males have been hindered by the lack of usable diagnostic tools for men or those adolescents associating with alternative gender roles.^14,15^ A large segment of the research on DE and ED research to date has been specific to elite athletes and focused on women. Thus, it is difficult to generalize findings to the entirety of the athlete population, although research concerning males is referenced in this paper.

In this paper, the authors will review the literature regarding adolescent athletes who intentionally engage in abnormal eating behaviors and focus on adolescent athletes of all training levels who may be affected by DE and ED.

Methods

In March 2019, a systematic literature search was completed by the authors in PubMed using the search terms "Feeding and Eating Disorders"[Mesh] AND (athlete OR athletes OR sport OR Sports) AND "high school.” Articles that did not explicitly mention and analyze high school or adolescent athletics were excluded (i.e., roughly 80%), although some of the additional research from non-athletes is referenced to provide readers with pertinent background information to understanding the phenomena of DE or ED.

## Summary of the Evidence

Twenty pertinent research articles were identified regarding adolescent athletes who may be at-risk for developing DE or ED. ED have been shown to impose higher rates of DSM-5 comorbidity than other psychological disorders and are frequently comorbid with each other. Only a small number of individuals with ED seek treatment.^16^ ED are more prevalent in adolescent elite athletes than non-athletes and can affect those identifying with all gender roles, all sport types and levels of competition.^17–19^

Additionally, adolescent athletes are more likely to demonstrate psychobehavioral facets of ED than adolescent nonathletes.^20^ Research concerning high school adolescent athletes has demonstrated mixed results regarding risk of ED development compared to non-athletes, but many studies agree that the perfectionist qualities of athletes may put them at increased risk.^21^ This perfectionism tendency can be a major contributor to athletes’ inaccurate belief that their body shape will be correlated with improved performance, a defining characteristic of DE.^22^

DE can lead to ED and individuals with DE are more likely to report mood disorders, such as depression.^23^ DE is known as one of the three pillars of the female athlete triad and is defined as abnormal eating habits.^24^ One of the primary causes of the female athlete triad is pressure from coaches and parents to improve performance by achieving and maintaining an unrealistic body weight and shape.^25^ In one study, 70% of students in both middle school and high school reported DE behaviors such as fasting, vomiting, and misuse of laxative and diet pills.^25^

Adolescence is a key period for the development of norms related to health behavior and body image and can be affected by social media such as Facebook, Snapchat, and Instagram.^26^ In the 2015 study by Carotte, Vella and Lim, a subgroup of 723 females were found to be 3.5 times more likely to follow at least one type of health and fitness-related social media content (OR 3.5, 95% CI 2.5-4.9, P < 0.001) and 2.4 times more likely to report having an ED if following such social media subject materials (OR 2.4, 95% CI 1.5-3.9, P < 0.001) than 278 males.^26^

Insufficient caloric intake can also cause a decrease in females’ estrogen levels and result in vaginal atrophy and amenorrhea.^27^ Menopause, which is pathophysiologically similar to amenorrhea, is associated with reduced endothelium-dependent dilation, a precursor to cardiovascular disease.^28^ Long term endothelial impairment has been shown to accelerate development of atherosclerosis in animal models and demonstrated to increase adverse cardiovascular events in humans.^28^ Additionally, experts have suggested that estrogen provides protection for the cardiovascular system in women, although this protection can diminish when estrogen levels are low.^28^

The lack of caloric intake from DE can result in electrolyte imbalances contributing to arrhythmias, gastrointestinal disorders, depression and suicidal thoughts.^29^ These types of electrolyte imbalances can influence the development of affective disorders.^29^ Chronic insufficient caloric intake generally has detrimental effects on adolescents’ bone development.

During adolescence, approximately 95% of a woman's total bone mineral density (BMD) is developed, with the largest BMD increase in BMD occurring between ages 11 and 14.^28,29^ If a female athlete’s body mass index (BMI) remains low (i.e., below 18.5 or below the 5th percentile) for an extended period, there can be an irreversible decrease in BMD.

This bone loss can result in a higher incidence of stress fractures and increased risk of osteoporosis.^30–32^ Combined with the high levels of physical activity many athletes experience, decreased BMD and resultant stress fractures can lead to decreased playing time. At the time of this literature review, there was little available evidence found concerning male athletes with ED and BMD changes.^32^

In fact, high school athletes who demonstrate DE behaviors take longer to heal from injuries than high school athletes who do not experience DE.^31,32^ Sports-related injuries can therefore subsequently increase their potential for obesity, hypertension, and diabetes during future years.^31^

In addition to primary care physicians and school nurses, coaches are often a potential first line of defense against ED and DE in athletes since they can observe a decrease in performance and increased rates of injury among their adolescent athletes.^33,34^ In a 2014 study of 123 high school coaches, 83% said that they were comfortable talking about DE with their female athletes, but only 40% reported having asked questions about abnormal eating patterns.^34^ The results of this study indicated that coaches can be instructed about what DE behaviors to look for and raise these sensitive issues with their athletes. In this same study, 69% of the coaches did not know that menstrual dysfunctions could be related to bone fractures in females, and only 24% reported having ever heard of the female athlete triad.^34^

In 2018, The National Collegiate Athletic Association reported that one of the best practices for mental well-being and resilience is the development of a health-promoting sports environment, advocating further education of coaches, students, and faculty athletic representatives.^35^ Of note, a 2012 study by Torres-McGehee, et. al. demonstrated that a random sample of 500 athletic trainers felt that they already had adequate nutritional knowledge to provide appropriate counseling to athletes.^36^

In the 2014 Kroshus, et. al. study of 370 high school nurses, less than a third reported awareness of the female athlete triad and less than 20% reported they could identify the three components of this triad.^37^ Although it was alarming that a smaller proportion of high school nurse respondents were aware of the female athlete triad, most nurses in the study were interested in the topic and wanted to learn more. In this same study, only 10.8% of the high school nurses reported their school had formulated specific policies to deal with ED.^37^ These results indicate that high school nurses could be provided information about the female athlete triad, ED, and DE since they could play a pivotal role screening for health risks and counseling at-risk student athletes.^35,37^

Early intervention has proven key for both prevention and treatment of ED.^38^ A 2017 intervention program study designed to increase athletes’ self-esteem showed that no new cases or symptoms of ED in the treatment group compared to the control group athletes.^39^

The results from a growing number of studies has suggested that female athletes and their families should be provided nutritional counseling and information about the female athlete triad and the potential long-term effects on overall health.^4,6,24,27^ In addition to nutritional counseling, however, theory-based intervention activities such as persuasive communication, active learning and observational modeling, can be helpful for reducing the use of restrictive dieting to decrease weight.^39^ Treatments should generally include daily calcium and vitamin D supplementation to counteract and prevent further bone loss.^30^

As indicated earlier in this paper, development of diagnostic tools for use with male athletes are still needed to better gauge those at risk for DE.^14^ Certainly, more general screening tools exist to help identify those athletes exhibiting DE behaviors. For example, the Eating Attitudes Test (EAT-26) is a tool developed by Garner et al. in 1979 to help identify individuals who may be at higher risk for DE.^40^ The EAT-26 has been shown to possess both test-retest and internal reliability (i.e., 0.75 to 0.88 at different grades)^41^ as a sensitive (i.e., 0.69), and specific (i.e., 0.93) for a cutoff score of 18.^42^ This instrument has also been translated into numerous languages.^41,43^ Other screening tools such as the Disordered Eating Attitude Scale (DEAS) have been less frequently evaluated than the EAT-26.^43^

## Conclusions

Based on this review of the published literature, we have concluded that adolescent athletes’ frequent drive to “be their best” combined with performance pressures from coaches and/or parents can frequently create an overly competitive environment contributing DE behaviors and ED. The long-term consequences of DE and ED are significant, including irreversible bone loss.^6,28,32^ Early detection is important, and thus primary care physicians, coaches and school nurses are frequently pivotal for identifying at-risk behaviors in adolescent athletes. Healthcare and school professionals need to be educated and trained to detect DE and the components of the female athlete triad.

Further DE research developments regarding interventions and screening methods for adolescent athletes interacting with healthcare clinicians of all gender identities are gravely needed. In addition to primary physicians, sports medicine physicians can also play a pivotal role to identify DE and ED in injured adolescent athletes. Due to their additional training and expertise, sports medicine physicians may in fact have more influence with adolescents who may be hesitant to report DE and ED. The professional literature to date emphasizes that education for all physicians, coaches, and nurses interacting with adolescent athletes can serve to prevent the significant morbidity and mortality associated with DE and ED.

### Conflict of Interest

The authors declare no conflict of interest.
